# A Functional Spiking Neural Network of Ultra Compact Neurons

**DOI:** 10.3389/fnins.2021.635098

**Published:** 2021-02-25

**Authors:** Pablo Stoliar, Olivier Schneegans, Marcelo J. Rozenberg

**Affiliations:** ^1^National Institute of Advanced Industrial Science and Technology (AIST), Tsukuba, Japan; ^2^Université Paris-Saclay, Sorbonne Université, CentraleSupélec, CNRS, Laboratoire de Génie Électrique et Électronique de Paris, Gif-sur-Yvette, France; ^3^Université Paris-Saclay, CNRS, Laboratoire de Physique des Solides, Orsay, France

**Keywords:** spiking neural networks, neuron models, leaky-integrated-and-fire, artificial intelligence, neuromorphic electronic circuits, neuromorphic computers, Jeffress model

## Abstract

We demonstrate that recently introduced ultra-compact neurons (UCN) with a minimal number of components can be interconnected to implement a functional spiking neural network. For concreteness we focus on the Jeffress model, which is a classic neuro-computational model proposed in the 40’s to explain the sound directionality detection by animals and humans. In addition, we introduce a *long-axon neuron*, whose architecture is inspired by the Hodgkin-Huxley axon delay-line and where the UCNs implement the nodes of Ranvier. We then interconnect two of those neurons to an output layer of UCNs, which detect coincidences between spikes propagating down the long-axons. This functional spiking neural neuron circuit with biological relevance is built from identical UCN blocks, which are simple enough to be made with *off-the-shelf* electronic components. Our work realizes a new, accessible and affordable *physical model platform*, where neuroscientists can construct arbitrary mid-size spiking neuronal networks in a *lego*-block like fashion that work in continuous time. This should enable them to address in a novel experimental manner fundamental questions about the nature of the neural code and to test predictions from mathematical models and algorithms of basic neurobiology research. The present work aims at opening a new experimental field of basic research in Spiking Neural Networks to a potentially large community, which is at the crossroads of neurobiology, dynamical systems, theoretical neuroscience, condensed matter physics, neuromorphic engineering, artificial intelligence, and complex systems.

## Introduction

The basic understanding of the dynamical behavior of Spiking Neural Networks (SNN) in Neuroscience is the focus of intense research. There are many relevant and pressing questions that are coming into focus, for instance, *breaking the neural code* what is the nature of the neural code? how information is encoded and transmitted with spikes from one part of the brain to another? how does that depend on network topology? are brain networks close to a critical or a chaotic state? what is the robustness of networks to chaos? how neurons may synchronize to form waves? how dynamical memories are realized and sustained? and many others (Korn and Faure, 2003; Rabinovich et al., 2006). These issues are being studied either by *in vivo* and *in vitro* experiments in neurobiology (Reyes, 2003; Yin et al., 2018), or theoretically by means of numerical simulations of mathematical models of neural networks (Izhikevich and Edelman, 2008; Stimberg et al., 2019). In the first case, the neurobiological experiments are technically challenging and, evidently, one cannot systematically modify the neural networks. In the second, the results on mathematical modeling may always be questioned, as a peculiar result that may depend on the assumptions made. For instance, it may be hard to assess if the relative ubiquity of chaotic behavior (Korn and Faure, 2003; Rudolph and Destexhe, 2007; Nobukawa et al., 2018) is of biological relevance. Moreover, in a well-known study, Izhikevich and Edelman (2008) observed the surprising result that the suppression of a single neuron out of an ensemble of a thousand spiking neurons may change the state of the entire network.

In order to better position our current work with respect to other approaches, we should also briefly describe the current efforts on applied research in Neuromorphic Computational Electronic Engineering for artificial intelligence (AI). That field can be roughly divided in two big areas. One aims to implement dedicated computer processors, which are optimized to run algorithms based on mathematical models of neurons. Examples of those *digital neuron* chips TrueNorth, Loihi, SpiNNaker, etc (Furber, 2016; Thakur et al., 2018). These systems are multi-core chips with *neuromorphic architecture*, that is, they have vast numbers of relatively small memory and processing units, which are densely interconnected. We may also include in this category the recent implementations using Field Programmable Gate Arrays (FPGA), which are making fast progress (Yang et al., 2015, 2018, 2019, 2020). On the other hand, a qualitatively different electronic engineering approach is aimed to design CMOS VLSI circuits, called *silicon neurons*, which implement the neuron models directly in hardware (Indiveri et al., 2011). Those neurons can then be interconnected *off-chip* to form networks by means, for instance, of the address event representation (AER) (Liu et al., 2015). In this approach, no actual spikes are transmitted between neurons, but it is the information of a firing event which is sent using the AER protocol between neuron addresses. One prominent example of a CMOS VLSI system is the BrainScaleS chip, which implement AdEx neurons (Schemmel et al., 2010) and further examples are discussed in Furber (2016); Thakur et al. (2018). In those approaches, the goal is to implement extremely large numbers of neurons (and synapses) to achieve the computing capacity of a brain. Some of their most significant challenges are to achieve low power dissipation and miniaturization. To reduce the power, one may work with transistors in the subthreshold regime (Mahowald and Douglas, 1991; Benjamin et al., 2014), however, additional issues arise in that case, such as device variability (Indiveri et al., 2011). Regarding the neuron circuit miniaturization, reducing its physical dimensions remains a challenge due to the relative large size of the capacitor that is required to represent the voltage of the membrane.

The limitations of silicon neurons may be potentially overcome by exploiting the neuromorphic functionalities of quantum materials (del Valle et al., 2018). For instance, phase change materials (Tuma et al., 2016), which accumulate phase instead of charge. Another notable example are the Mott insulators, whose memristive properties permit the firing of an electric spike when they are driven across the insulator to metal transition (Janod et al., 2016). Despite intense activity, this field of research is emerging and currently proposed devices are still individual *single* neurons (Stoliar et al., 2017; Yi et al., 2018). The main challenges ahead are to achieve a reliable control of the materials and the understanding of their fundamental physical behavior (del Valle et al., 2019), before actual circuits may be implemented.

In the context of the approaches that deal with SNN systems that we described above, our present methodology shares features of many but is qualitatively different to all of them. The main goal of the present work is to bring to the research community a novel way to build and study SNNs of unprecedented simplicity, which opens a new way to do experimental work in basic neuroscience. We propose a methodology to build general neural circuits of *a priori* arbitrary topology, where spiking neurons are in direct interaction and are interconnected as *lego*-like blocks. To illustrate this point, we shall adopt a classic model of neuroscience: the Jeffress model of binaural detection of sound directionality (Jeffress, 1948).

Our methodology is built around a recently introduced electronic neuron circuit, the ultra-compact neuron (UCN) (Rozenberg et al., 2019). This UCN, like Mott neurons exploits a memristive behavior, however, in contrast to those it is not based on a quantum material but on a conventional electronics device, the thyristor. So in this regard, the UCN may be considered a silicon neuron model. The silicon neurons vary in their degree of circuit complexity, for instance realizations of the AdEx model may require tens of transistors (Indiveri et al., 2011). The UCN, in contrast, counts with a small number of components so it can be considered a *compact neuron* model, following the terminology introduced by Indiveri et al. (2011). Moreover, the UCN can be termed *ultra-compact* as it requires a *minimal* number of components. In fact, to realize a basic circuit of a leaky-integrate-and-fire (LIF) neuron model, the UCN requires, like most other silicon neurons, a capacitor to *integrate* charge, and a *resistor* to mimic the *leaky* feature. However, the novelty of the UCN is that the *fire* functionality can be realized by a single silicon controlled rectifier (SCR or thyristor), which is a conventional electronic device introduced in 1956. The key insight is to realize that the I-V characteristics of the SCR display memristive features analogous to that of Mott materials, which enable the neuromorphic electric spiking behavior. However, the SCR present the advantage that they are available *off-the-shelf* to implement artificial neurons (Rozenberg et al., 2019), avoiding the need to deal with the complexities of Mott quantum material fabrication and control. We should also mention that the UCN is not restricted to the LIF model as it can be easily extended to realize a large variety of biologically relevant spiking neuron models (Stoliar et al., 2020).

We should make clear that the present UCN based electronic circuit methodology is not aimed at competing with engineering implementations for AI, therefore, speed, dissipation, and physical size is not our immediate concern. Here, we explicitly demonstrate that one can make a crucial step up and go from the level of a single UCN spiking, to a functional and biologically relevant circuit of more than ten neurons. One qualitative difference with respect to neurocomputing engineering implementations is that the UCN network operates in real continuous time, with time scales in principle compatible with those of biological systems. The aim of our work is a proof-of-concept for a novel, flexible, and affordable platform to construct spiking neural networks. This methodology can be further scaled up to hundreds or possibly thousands of neurons, which can be implemented with low-cost printed circuits boards (PCB). This order of magnitude in the number of neurons is sufficient to study questions of basic neuroscience, such as those that we mentioned in the beginning. In other words, we aim at opening a new experimental field of basic research in Spiking Neural Networks to a potentially large community, which is at the crossroads of neurobiology, dynamical systems, theoretical neuroscience, condensed matter physics, neuromorphic engineering, artificial intelligence, and complex systems.

The paper is organized as follows: We shall first describe the basic features of the UCN circuit and of the Jeffress model SNN. We shall then show how UCN blocks can be used to implement the network, proceeding in two steps: Firstly, we shall construct a novel *long-axon neuron*, which will serve as the delay-line of the Jeffress model. Secondly, we shall combine two such long-axon neurons with a layer of output neurons that detect the *coincidence* of spikes propagating along the delay lines. We shall then demonstrate the neuro-computational functionality of sound directionality detection. Finally, we discuss the prospects of adopting the UCN as a general and easy to implement spiking neuron *lego*-like block for experimental electronic neuroscience and neuro-engineering.

## Model and Methods

### The Ultra Compact Neuron Circuit

The UCN circuit can display electric spiking behavior analogous to a biological neuron and constitutes the basic building block of our methodology. In (Rozenberg et al., 2019) we demonstrated a key feature of UCN, namely, that one (upstream) UCN block can be directly connected to a second (downstream) UCN block, and that the spiking behavior in the former can elicit spiking behavior in the latter. In the present work we go beyond, and show that arbitrary *functional circuits* can be implemented, rather straightforwardly, using the UCNs as a *lego*-like constructive blocks without any need to use of AER protocols. Our approach should be viewed as a novel, simple and flexible *experimental* platform for neuroscience research in SNN, alternative and complementary to numerical simulations, *in vivo* and *in vitro* biological studies, and electronic engineering for artificial intelligence applications. We shall next briefly describe the basic behavior of the UCN, for the sake of completeness. Further details can be found in Rozenberg et al. (2019), Stoliar et al. (2020).

**FIGURE 1:**
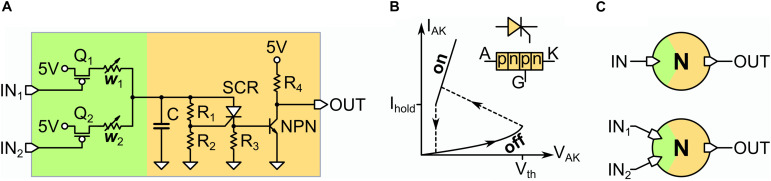
**(A)** Electronic circuit of the UCN unit. The input (green) is shown for the case of two dendrites. In orange we depict the section of the circuit where the action potentials are generated (see text for description). **(B)** Schematic I-V characteristics of the SCR and electronic symbol of the component. Note the hysteretic behavior similar to that observed in VO_2_ Mott neurons (del Valle et al., 2018). **(C)** Symbol of the artificial neuron for the case of a one-input **(top)** and two-input **(bottom)** UCN, which are used as *lego*-like building blocks (the list of the electronic components is provided in the Supplementary Material).

In Figure 1, we show the electronic circuit of the UCN (Rozenberg et al., 2019). The UCN is a minimal physical implementation of a leaky-integrate-and-fire model that generates action potentials. This model is minimal since the key part of the circuit, which generates the action potentials, only requires three basic elements (see orange region in Figure 1A). Similar to most silicon neuron implementations (Indiveri et al., 2011), it has a membrane capacitor (C) for the integrate function, a resistor (R_1_ + R_2_) for the leaky function. However, in contrast to conventional CMOS implementations, the fire function is realized by a SCR. The fire function is most easily understood by noting that the SCR, which is a *pnpn* device, can be considered as a diode with a threshold (see Figure 1B). Thus, it is normally off with a large resistance R_*HI*_, bigger than leak resistors (R_1_ + R_2_) so that when current inputs the neuron, the capacitor gets (leaky) charged, with time constant τ = (R_1_ + R_2_)C. The fire occurs when the SCR suddenly commutes to the low resistance value R_*LO*_. This happens when the SCR gate voltage reaches the threshold value V_*th*_, which is a parameter of the SCR. Thus, the fire event can be easily tuned with the resistive voltage divider, such that the condition V_*th*_ = V_*c*_ R_2_/(R_1_ + R_2_) is met. Or in other words, this condition sets the critical value that V_*c*_ needs to reach so that a spike is generated. At this point the resistance of the SCR collapses, as its *pn* diode-like junctions become forward polarized. Since the SCR resistance becomes much smaller than the leak pair (R_1_ + R_2_), the membrane capacitor rapidly discharges through it, producing a spike of voltage, or action potential, on the small resistor R_3_. The time-scale for the duration of the spike is ∼ (R_3_ + R_*LO*_)C. The action potential terminates when the current spike decreases beneath a value I_*hold*_, which is another parameter of the SCR. Thus, we see that this hysteresis in the resistance, or memristive property, of the SCR is the key feature behind the simplicity of the UCN.

Finally the action potential spike needs to be strengthened so it can drive a downstream UCN connected to its output. This is simply achieved by a transistor and a voltage source. The green region in Figure 1A depicts the synaptic connections or dendritic inputs of the neuron. For the Jeffress model implementation we shall only need either one or two inputs (see Figure 1C). In the Supplementary Material we also provide further details on the circuit implementation of the UCN units, along with the list of *off-the-shelf* electronic components. Importantly for the sake of adopting the UCN units as a physical platform for arbitrary SNNs, we also present simulations on their fan-in/fan-out performance.

### The Jeffress Model for Detection of Sound Directionality

We shall consider the Jeffress model (Jeffress, 1948) as a prototypical SNN with a biological functional to show how such a system can be implemented with the UCN blocks that we just described. It is interesting to mention that this model was also the focus of the pioneering work in neuromorphic engineering by Lazzaro and Mead (1989), though more than 30 years ago and following a qualitatively different implementation based on CMOS. Subsequent work along those lines was done by Bhadkamkar and Fowler (1993) who developed a CMOS chip with about 50 thousand transistors working in the subthreshold regime (Bhadkamkar, 1994). These lines of work are and illustration of the electronic engineering approach and the challenges that subthreshold systems may present, as described before.

The Jeffress model was introduced in 1948 to describe the brain’s neural system for detection of sound directionality on the azimuthal plane (Jeffress, 1948). It makes a mapping of sound angle to neuronal space location. This is achieved by exploiting the time difference of the arrival of sound to the ear, and translating that difference into neuronal location by means of long-axon neurons that act as homogeneously graded delay lines. Neurons, which receive inputs from these graded axonal lines, act as coincidence detectors which fire when spikes simultaneously arrive from the two sides (Konishi, 1993). Remarkably, the relevance of the Jeffress model has survived the ongoing revolution in experimental neuroscience (Joris et al., 1998; Burger et al., 2011; Cariani, 2011). Moreover, it has received some striking validations of its most basic aspects through the neuronal mapping of the auditory system of birds (Young and Rubel, 1983).

**FIGURE 2:**
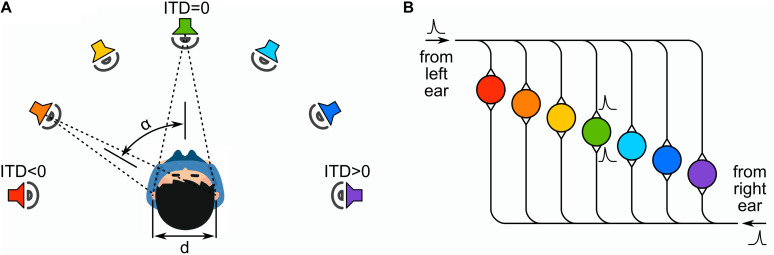
**(A)** The sound is incident with an angle α on the azimuthal plane that is transduced into an interaural time difference (ITD) for the excitation of the left and right ears auditory system. **(B)** The system generates spikes at the L and R input neurons that propagate down their respective long axons (delay lines). The ITD is mapped onto the position of output neurons that detect the coincidence of spikes propagating down the long axons. Two spikes arriving in coincidence to the green neuron when α = 0 and ITD = 0.

In Figure 2 we show a schematic diagram of the Jeffress model of binaural detection of sound direction. The sound is assumed to originate along the azimuthal direction, thus depending on the incidence angle the sound-wave front will arrive with an interaural time difference (ITD) to the ears (Figure 2A). The front arrival at the left (L) and right (R) auditory systems provokes the excitation of the respective input neurons. These neurons send spikes that propagate down their long-axons, similarly as a signal down a delay-line (Figure 2B). An layer of output neurons detects the (time) coincidence of the propagating spikes. Thus they provide a neurocomputation or transduction of the incidence angle into an electric signal encoded by the excitation of the output neuron array.

As one can see from the diagram of Figure 2B, the Jeffress model has two type of neurons: the input neurons are characterized by a long axon and the output neurons have two dendritic inputs to detect simultaneous spikes (i.e., coincidences) occurring a two given points on the L and R axons.

### The Long-Axon Neuron

To implement the long-axon neuron circuit it is useful to recall that the celebrated Hodgkin-Huxley (HH) model was introduced to precisely describe the propagation of an action potential along the axon of a neuron (Hodgkin and Huxley, 1952). It was formulated as a set of differential equations of classic cable theory. The parameters of the equation are the voltage-gated conductance and capacitances per unit length. The non-linear dependence of the ion channels conductance with the membrane potential leads to the local action potential generation in a given segment of the axon. The propagation of the signal follows from the fact that one action potential spike at one axon segment induces a successive spike in the neighboring segment. The propagation is initiated in the axon hillock of the cell soma, which is the location where the soma connects to the axon. In this manner, the spike signal travels down the axon at a constant speed.

Since in each segment an action potential is generated, we may use one UCN to describe each segment, and then build the axon simply by means of a chain of UCNs. The key feature is that the parameters of each UCN have to be chosen such that a single spike of a given segment is sufficient to reliably elicit a spike in the subsequent neighboring segment (Rozenberg et al., 2019).

**FIGURE 3:**
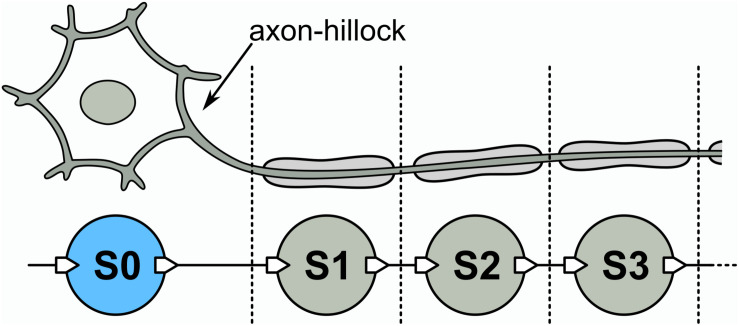
**Top:** Schematic long-axon neuron. **Bottom:** Implementation of a long-axon neuron by a uni-dimensional network of UCN units. S0 is the soma + axon-hillock, S1, S2, … are axon segments. The list of components of the UCN blocks are provided in the Supplementary Material.

From the previous discussion, we may now introduce a long-axon neuron model that emulates the mechanism of spike propagation described before. The model is schematically shown in Figure 3. It has an initial UCN unit that represents the soma-plus-axon-hillock (S0), followed by a succession of UCN units that represent the segments of the long axon (S1, S2, S3,…). As in the HH model of a neuron axon, each one of those blocks generates an action potential and induce a subsequent one on the neighboring downstream segment. Thus, the long-axon neuron is built as a one dimensional chain of spiking UCN units. For simplicity, we adopt all the UCN blocks to be identical and set to fire with just one single incoming pulse. However, it would be easy to adapt the first “soma” block S0 to fire a spike upon any other input signal of our choice.

### The Jeffress Model SNN Circuit

**FIGURE 4:**
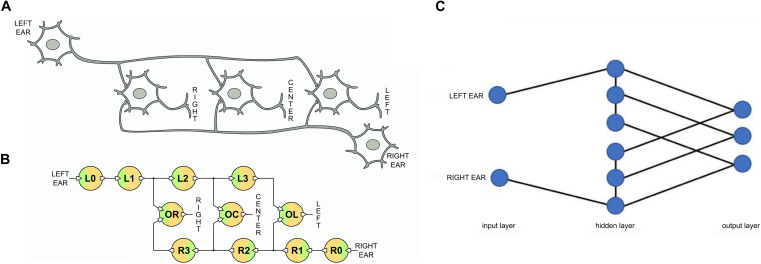
**(A)** Schematic neuron network of the Jeffress model. **(B)** Corresponding spiking neural network implemented with UCN blocks. **(C)** SNN architecture. The Jeffress model is neither a simple feed-forward nor a recurrent neural network due to the intra-layer connections. Also notice if the hidden layers were redefined, there would be connections toward the output layer that need to skip layers.

We can now use the long-axon neurons to implement the neural network of the Jeffress model that we show in Figure 4. We adopt one long-axon neuron to represent the left ear input and another one for the right ear input. In the former the spikes propagate from left to right and in the latter in opposite direction. In addition, we need to interconnect the long axons of these input neurons with an array of output neurons that detect the coincidences. We implement the output array also using UCN units, as shown in Figure 4. In this case the UCN blocks are tuned to fire upon arrival of two overlapping input signals, i.e., to detect coincidences of pulses at their two dendritic inputs. The three output neurons depicted in the diagram of Figure 4 are labeled “left,” “center,” and “right” as they encode for sound signals incoming from those respective sectors.

The scheme of the circuit can be easily scaled up to increase the system’s resolving power of the azimuthal angle. In the Supplementary Material we provide an explicit realization scaling up the number of UCNs in a long-axon neuron by means of a realistic numerical simulation. However, in the following we shall restrict ourselves to implement a small network where the behavior of each neuron can be individually traced.

In principle, a transducer, such as the cochlea in humans, converts the incoming sound-wave front into input excitations to the network. The interaural time difference of the wave front results in a time delay between the spikes generated by L0 and R0. These two “soma + axon-hillock” neurons may, in principle, be setup to fire upon any desired excitation threshold, such as depending on the intensity level, frequency, etc. Here, again for the sake of simplicity, we set them to fire upon arrival of a single above-threshold voltage pulse.

Finally, we may also mention that from a SNN architecture point of view, the Jeffress model is neither a simple feed-forward nor a recurrent NN. This can be appreciated in Figure 4C, where we show the network architecture as layers. We can observe that unlike feed-forward networks there is communication within a given layer (the middle one), and also unlike recurrent networks, there are no connections going backward.

## Results

**FIGURE 5:**
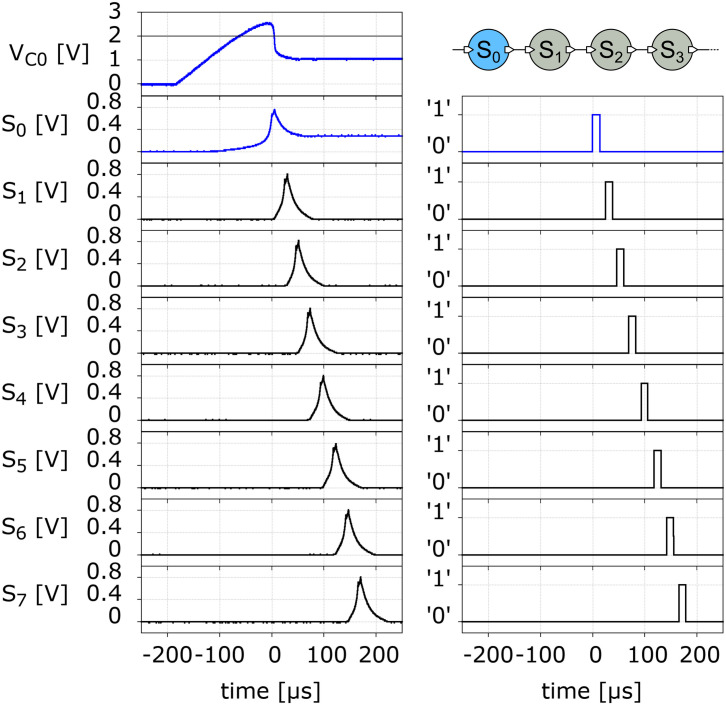
**Left panels:** Measured action potential spike signal propagating along a 7-segment long-axon neuron **(top right panel)**. The **top left panel** V_*C*0_ has the voltage on the membrane capacitor C (see Figure 4). The signals S_0_ to S_7_ correspond to the action potential spike voltage measured on the resistor R_3_ at the exit of the SCR (see Figure 4). The corresponding panels on the right have the output of the respective neurons. The logic “0” corresponds to a physical voltage value of 5 V, and the “1” to 0 V.

We now turn to describe the behavior of our neuromorphic model. We begin with the long-axon neuron. In Figure 5 we show the experimental data of the spike propagation along the axon. We observe that a spike travels down the axon as each pulse at a given segment unit induces a subsequent pulse at its downstream neighbor. For the chosen values of the circuit components (see Supplementary Material) the pulse moves along the axon at a constant rate of approximately 0.045 segment/μs. This rate may be further increased by decreasing the capacitance of the UCN.

**FIGURE 6:**
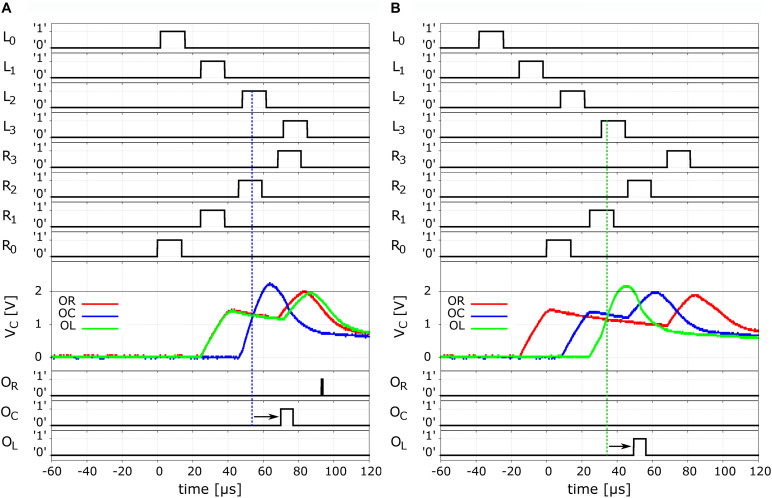
Neurocomputation of ITD. **Left panels (A)** ITD = 0 μs and **right panels (B)** ITD = 40 μs. Top four rows show the traces of the propagation of the spikes along the right-ear delay line, from R0 to R3, measured at the respective unit outputs. The next four lines show the respective signals for the spikes propagating in the left-ear delay line, in opposite direction. The mid panel show the membrane voltage VC for the three output neurons. As expected for the symmetric case of ITD = 0 μs (α = 0), we observe the build-up of the potential of the central neuron OC (blue). When this membrane potential overcomes the threshold it sets off an output spike as can be seen in the trace of the output neurons in the last three rows, where we indicated an additional delay by the small arrows. The right hand panels have the respective traces for the case of a finite ITD = 40 μs, where we observe that the coincidence was detected by the OL neuron (green).

We may now present the experimental data for the full Jeffress model implementation. This is shown in Figure 6, where we show two examples of the system’s behavior upon arrival of an input to the left and to right ears, with a given ITD. We shall begin with the case of no delay between arrivals, i.e., ITD = 0. This is shown in the Figure 6A. We can observe that the action potentials propagate through each long-axon neuron in opposite directions and at the same speed (first and second panels from the top). Indeed, they arrive to the last segment of each long-axon at about the same time 80 μs. The small differences between the timings and propagations between the two long-axon neurons is due to the small variability among electronic components, which is specified by the maker. An important point to make here is that the representation of time in SNNs is a non-trivial task (Liu et al., 2015). It usually requires off-chip communication and an AER protocol. In contrast, our circuit implementation is operating directly in real continuous-time. Moreover, the time-scales of our model are compatible with biological neuron networks ones.

As shown in the diagram of Figure 4, we see that the detection of coincidences is conducted between the UCN pairs L3-R1, L2-R2, and L1-R3. From the data in Figure 6A we can see that the “crossing” of the propagating spikes is, as expected, between the second segment of each long axon, namely, the pair L2-R2. The traces of the membrane potentials of the output neurons is shown in the third panel from the top of Figure 6A. We see that the corresponding “center” neuron senses the arrival of the pulses in coincidence and consequently increases its potential that overcomes the threshold of ∼ 2 V and fires. The fire event of the output neuron OC that signals the detection of sound from the central quadrant can be seen in the bottom panels of the Figure 6A. We see a strong spike in the trace of OC, however, another smaller one can also be observed for OR. These latter one can be considered a residual effect that are rather natural to this system’s bio-mimetic neural architecture. We will come back to this point later on.

In the panels of Figure 6B we show the case of a finite delay arriving to the L and R ear input neurons. As can be seen in the top panels, the approximate delay is of 40 μs, arriving earlier at the “left” ear L0, which indicates that the sound would have come from the left sector.

From the propagation of the spikes depicted in these panels, we observe that coincidence is occurring between UCN pair L3-R1, which is detected by the output neuron OL. In this case, the third panel shows the membrane potential of “center” and “right,” and we see that they detect the passing of spikes through the long-axons but not in coincidence. Hence their voltage initially increases and then starts to leak. By the time the second pulse is detected, although the V_*C*_ has not yet fully relaxed, the new increase is not enough to reach the threshold. The V_*C*_ fall just short of overcoming the 2 V value. Thus, the sole coincidence is detected by the “left” output neuron OL, as can be seen in from its firing event in the bottom panel of the Figure 6B.

Comparing the input spikes and the output fire, we may notice the existence of a small delay, which is indicated in the traces of the output neurons OC in Figure 6A and of OL in Figure 6B with small arrows. The delay is of a few microseconds and is simply due to the time it takes for the output neuron to integrate the coincident input signals at its dendrites and fire, namely, to perform its neurocomputation.

**FIGURE 7:**
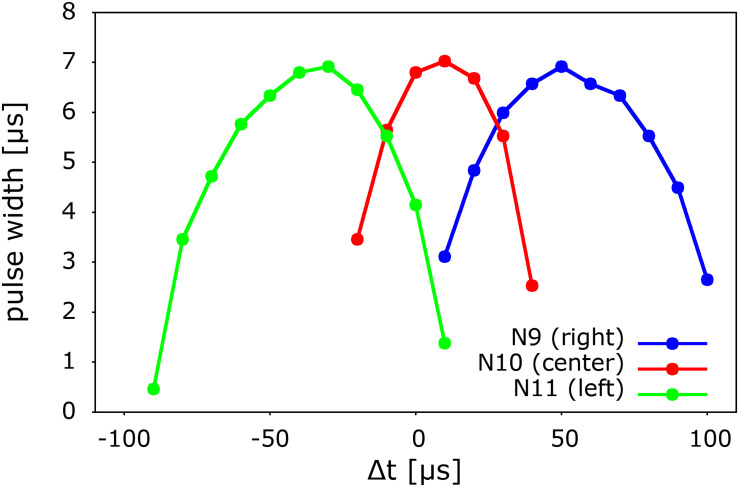
Systematic variation of the signals of the three output neurons of the model as a function of the incoming time delay of ITD. The result of the system’s neurocomputation is coded in terms of pulse width, which is also the intensity of the spike generated by the output neurons. Note (Figure 6A) that the pulse width indicates the degree of overlap or coincidence of the propagating spikes. The delay Δt is defined as the time difference between the pulses at L0 and R0. A positive Δt corresponds to a pulse generated on the right side. For simplicity, the pulses were generated with a pulse generator.

In Figure 7 we finally show the systematic behavior of the output as a function of the time difference between the arrival of pulses to the left and right “ears” (L0 and R0) or ITD. We observe and approximately left-right symmetric behavior of the three output neurons with respect to the arrival delay. The output is computed and coded in terms of the pulse width of the output signal, as was indicated before in the case of the central sound incidence, or ITD = 0, shown in Figure 6A. From this type of coding we observe that similarly as in biological systems, there is a continuum of detection and we adopt the strongest signal (winner takes all) as the result of the neurocomputation.

As it can also be seen in Figure 7, the resolution of the time difference of our system is about 40 μs, which corresponds an azimuthal angular of about 4°, assuming an ear-to-ear distance of 0.2 m and 343 m/s for the speed of sound (Glackin et al., 2010; see Supplementary Material). In our present simple implementation, the angular resolution depends on the time it takes for a spike to propagate along two subsequent Ranvier nodes of the long-axon neuron (see Figure 5 and Supplementary Material). This timescale depends on the specific values of the UCN parameters. For instance, a bigger capacitor would require more time to get charged up to the fire threshold value, thus would increase the time it takes the spike to travel down the long axon. Hence this would decrease the angular resolution of the system (see Supplementary Material). Conversely, the resolution can be systematically improved by performing faster integration, which can be achieved using smaller capacitors for the membrane potential. Therefore the resolution depends on the specific values of the circuit components of the UCN blocks of the long-axon neuron (Rozenberg et al., 2019). Note, however, that given a delay resolution, the maximal angle that the model may detect depends on the number of Ranvier nodes implemented in the long-axon. In the Supplementary Material we provide details on how to implement a large sound detection angle.

Finally, from the data of Figure 7 we may also notice that the symmetry of the response is affected by a small off-set shift of about 10 μs. This is simply due to the fact that the SCRs, as all electronic components, have a small dispersion, or tolerance, in their production. This feature can be easily corrected by a feed-back loop of the output, which may tune one of the resistors at the gate of the SCR that sets the firing threshold. This correction can be cast in terms of a *supervised* learning of the auditory system, where a correction pulse generated by the “wrong” output may tune the value of a *memristor* at the gate of the SCR. Details of that procedure are described in the Supplementary Material.

## Discussion

We have shown how a functional spiking neural network of biological relevance and be constructed using the recently introduced ultra-compact neurons as building *lego*-like blocks. The specific neuronal network is the Jeffress model of the auditory system of animals and humans, which remains a central paradigm in the field more than 70 years after its original proposal. This model was also adopted by Lazzaro and Mead (1989) in their pioneering work to demonstrate the concept of neurocomputing.

To implement the Jeffress model, we have first introduced a novel *long-axon* neuron, which we motivated by analogy with the Hodgkin-Huxley model for axon propagation of action potentials. Our long axon-neurons were built as a chain of UCN units, where the initial one plays the role of a soma + axon-hillock. The spikes then propagate along a chain of UCN segments forming a transmission line of action potentials, analogous to the nodes of Ranvier of biological axons. Two of such long-axon units constitute the *input* neurons that run antiparallel to each other. They are interconnected via an output layer of two-dendritic inputs neurons, which are also implemented by UCN blocks. The result of the neuro-computation is coded by the spike intensity of the output neurons.

The present work is a concrete demonstration of how the UCN is a simple and versatile (Hodgkin and Huxley, 1952) building block that allows to construct bio-inspired spiking neural networks. This simplicity allows the UCN networks to be readily made from *off-the-shelf* electronic components. We should emphasize the important feature that the UCN is a *physical model* of a spiking artificial neuron that does not include unphysical features such a hard voltage reset nor an abstract spike signal. Those artificial characteristics are indeed present in the integrate and fire mathematical models and are a source of concern for the stability of the dynamics (Korn and Faure, 2003; Nobukawa et al., 2018).

One may envision that neuroscientists may exploit the simplicity and convenience of the present scheme in multiple manners. For instance, they may begin to build physical SNN of UCNs to address under a new light open issues, such as the nature of the neural code in feed-forward and recurrent neural networks. So far, the study of these important questions has essentially been restricted to numerical simulations of schematic mathematical models and algorithms.

The present work introduces a novel experimental platform for the systematic construction and study of the dynamical behavior of SNN. This platform is simple, versatile and affordable, so it may open a new avenue of research in experimental neurobiology at a large and exciting interdisciplinary crossroad.

## Data Availability Statement

The original contributions presented in the study are included in the article/Supplementary Material, further inquiries can be directed to the corresponding author.

## Author Contributions

All authors listed have made a substantial, direct and intellectual contribution to the work, and approved it for publication.

## Conflict of Interest

The authors declare that the research was conducted in the absence of any commercial or financial relationships that could be construed as a potential conflict of interest.
