# Supplementary material for: A Functional Spiking Neural Network of Ultra Compact Neurons
Source: Front Neurosci. 2021 Feb 25;15:635098. doi: 10.3389/fnins.2021.635098 (PMC7947689; doi:10.3389/fnins.2021.635098)
Supplement: Supplementary file 1 [file Data_Sheet_1.docx]

Supplementary Material

# Simulation of fan-in and fan-out of the UCN

By means of simulations, we show that one UCN may have more than 100 parallel dendritic inputs, without any inconvenience. Similarly a single UCN may drive more than a 100 downstream UCNs without any degradation in performance.

## Fan-in of the UCN

**Supplementary Figure 1.** Circuit of 120 parallel dendritic inputs driving 1 downstream UCN neuron. The input neurons are excited with a single pulse each, that is generated at a random time in the interval 0-20ms

**Supplementary Figure 2.** Simulation results of the previous circuit. One may observe that the membrane capacitor voltage (VC) and the output voltage (VOUT) of the downstream UCN vary without any inconvenience and as expected for the random inputs.

## Fan-out of the UCN

**Supplementary Figure 3.** Circuit of 1 UCN driving 100 parallel downstream UCNs. Note that we adopted N01 and N02 as two different neurons. They have the same circuit, they simply are chosen to differ in their time-constant as N01 has a 50nF capacitor and N02 a 100nF one. There are fifty N01 neurons and fifty N02 in the simulation.

**Supplementary Figure 4.** Simulation results of the previous circuit. One may observe that the output voltages of 2 types of downstream UCN show thir spiking behavior without any degradation in performance.

# Long-axon neuron circuit made of a cascade of 50 UCN neurons

**Supplementary Figure 5.** Circuit of a long-axon neuron (delay line) made of a cascade of 50 UCNs. Each UCN is indicated by the symbol as in T1H. They are all identical with identical component. The values of the components are indicated in the circuit of the 3rd panel.

**Supplementary Figure 6.** Simulation of the long-axon neuron with 50 UCNs using LT-Spice: One may observe how the spike is propagating down the long-axon neuron as in a delay line. For simplicity we only show the first and the last 5 neurons. These simulation results show that there is no problem to upscale the circuit of the long-axon-neuron to an arbitrary number of UCNs

# Jeffress model with two long-axon neurons of 22 UCNs each plus 21 coincidence-detecting output layer UCNs

In the circuit below, we show how the resolution for the azimuthal angle detection can be simply improved in our Jeffress model implementation. This is achieved by simply increasing the number of Ranvier nodes in the long-axon neuron, and simultaneously increasing the number of output neurons that detect spike coincidences. In the main text of the article we, for the sake of clarity we just used three UCN to model three Ranvier nodes, it therefore allowed the system to resolve three directions, left, center and right. The angle resolution is directly related to the number of UCNs conforming the long-axon neuron. We shall show below the detailed circuit to achieve increased angular resolution, and the corresponding simulation results using LTspice. In Fig.S7 we show the full circuit, which simply amounts to increase the number of UCN blocks in the long-axon neuron and the corresponding number of coincidence detection neurons in the output layer (see Fig.4 of Main Text). We adopt 21+1 UCN for each one of the two long-axon neurons (21 for the Ranvier nodes and 1 for the soma), and 21 UCN with two inputs (see Fig.1 of Main Text) for the coincidence detection.

In Fig.S8 and S9 we provide the explicit circuits for the UCN blocks, which include the values of the electronic components used in our simulations.

**Supplementary Figure 7.** Circuit of two long-axons of 22 UCN each, and 21 coincidence UCN


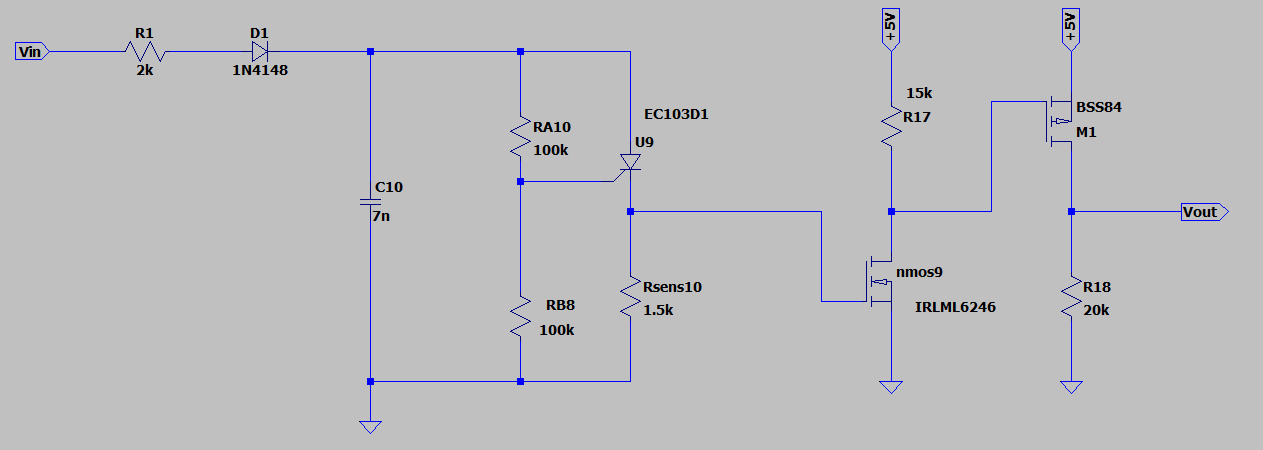


**Supplementary Figure 8.** Detail of circuit elements of each UCN forming the *cascade* of the long-axon neuron, which includes the values of components.


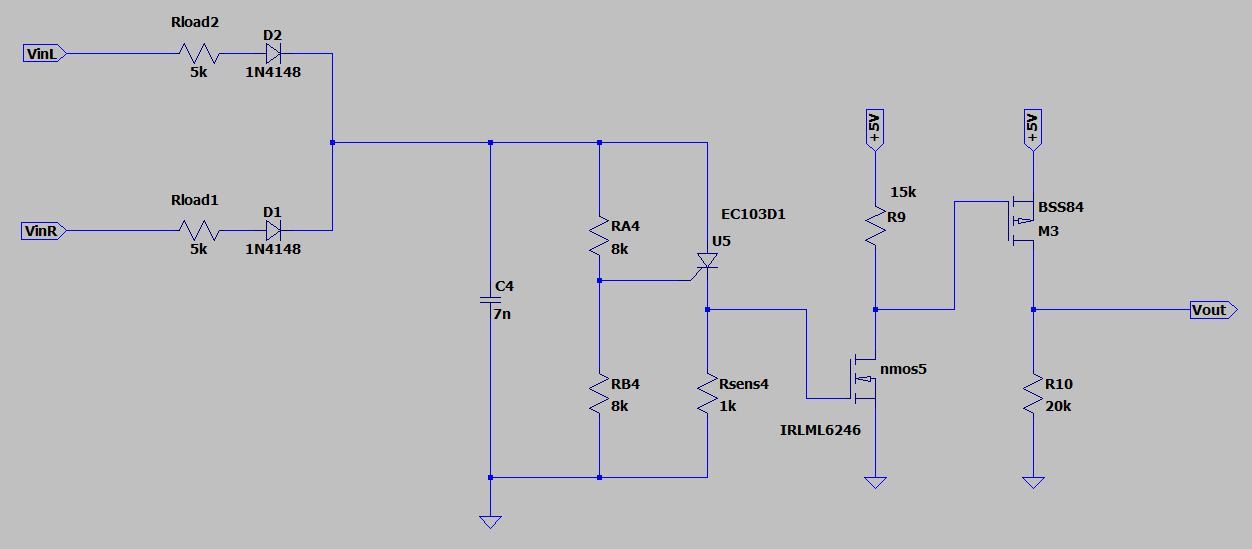


**Supplementary Figure 9.** Circuit of a *coincidence* neuron, which includes the values of components

In Fig.S10 we show the results of the simulations. The x-axis has the interaural time delay between the input pulses arriving to the left and right soma of the long-axon neurons. On the y-axis we indicate which of the 21 output neurons fires most strongly (see Figs.6 and 7 of Main Text). Neuron #11 indicates the center (delay = 0μs)

To relate interaural time delay (ITD) to azimuthal angle (θ) we can follow Glackin et al [1] and use Rayleigh's formula

ITD = r/c [θ + sin(θ)]

which for a head radius r = 0.1m and taking the velocity of sound c = 343m/s, gives an angle θ of 4° for an ITD of 40µs. For a right angle it gives the maximal delay ITD = 750μs. Therefore, for our system with a maximal delay of 400μs, we are approximately spanning a 45° angle, thus an azimuthal angular resolution of about 4°-5° as indicated before.

**Supplementary Figure 10.** Simulation results with LT-Spice, of coincidence detection by output layer neuron: Data shows the # of the output UCN that fires as a function of the input delay ITD. The results of the simulation do not show any degradation of performance upon upscaling of the number of UCN in the long-axon neurons

[1] B. Glackin, J. A. Wall, T.M. McGinnity, L.P. Maguire and L.J. McDaid, "A spiking neural network model of the medial superior olive​ ​using spike timing dependent plasticity for sound localization" Front. Comp. Neurosci. 4, 1 (2010)

# Off-line learning of a non-ideal circuit (due to parasitic delay)

## Circuit and simulation of the 2 long-axon and 3 coincidence neurons system

**Supplementary Figure 11.** Circuit of the 2 long-axon and 3 coincidence neurons system. The components values are the same as in the circuit section 2.

**Supplementary Figure 12.** Simulation of the circuit using LT-Spice; Low frequency input pulses (20µs duration, 6ms period) are applied simultaneously to the system inputs (IN left and IN right), i.e. with ITD = 0μs. Hence the coincidence neuron that codes for center direction NC fires when the spikes travelling along both axons reach simultaneously NC. Neither NR nor NL fire.

## Simulation of the circuit when a “parasitic” delay exists at the right input

**Supplementary Figure 13.** Simulation of the circuit using LT-Spice when a “parasitic” delay exists at the right input. Here, a parasitic delay has been introduced at the first axon neuron to which IN_right is applied. This is practically obtained by increasing the value of the capacitance of the corresponding UCN from 7nF up to 17nF. Hence, as illustrated below, such parasitic delay leads to the “erroneous” result of the neurocomputation as neuron NL spikes instead of NC.

## Feedback loop circuit to self-correct the parasitic delay

The circuit is the same as the previous one, except that one of the UCN sections belonging to the top long-axon neuron connected to IN_left (see figure hereafter) has been modified. In such neuron, the resistance connected across the SCR gate and ground, corresponds now to a memristor U12, whose resistance can vary from 100kΩ downto 26kΩ, as a decreasing function (proportional to the-tanh function) of the number of output spikes of NL. Notice that for simplicity in the LTspice simulation we defined the memristor as a three-terminal device, while they often have only two. However, in order to tune the value of the resistance one needs to add an additional selector circuit that allows to disconnect the memristor from the circuit so tuning pulses may be safely applied. We may omit the selector by introducing a fictitious third electrode, which plays an analogous role to tune the resistance.

**Supplementary Figure 14.** Feedback loop circuit to self-correct the parasitic delay

Hence in the simulation below, NL spikes due to the parasitic delay, but U12 decreases progressively, yielding a local increase of the delay time in the top axon: after a few IN_left and IN_right incoming pulses, the system has autocorrected itself, as illustrated below : NL does not fire any more, and NC has started firing.

**Supplementary Figure 15.** Simulation of the feedback loop circuit to self-correct the parasitic delay

# Implementation of the UCN blocks

## Circuits used for the neurons


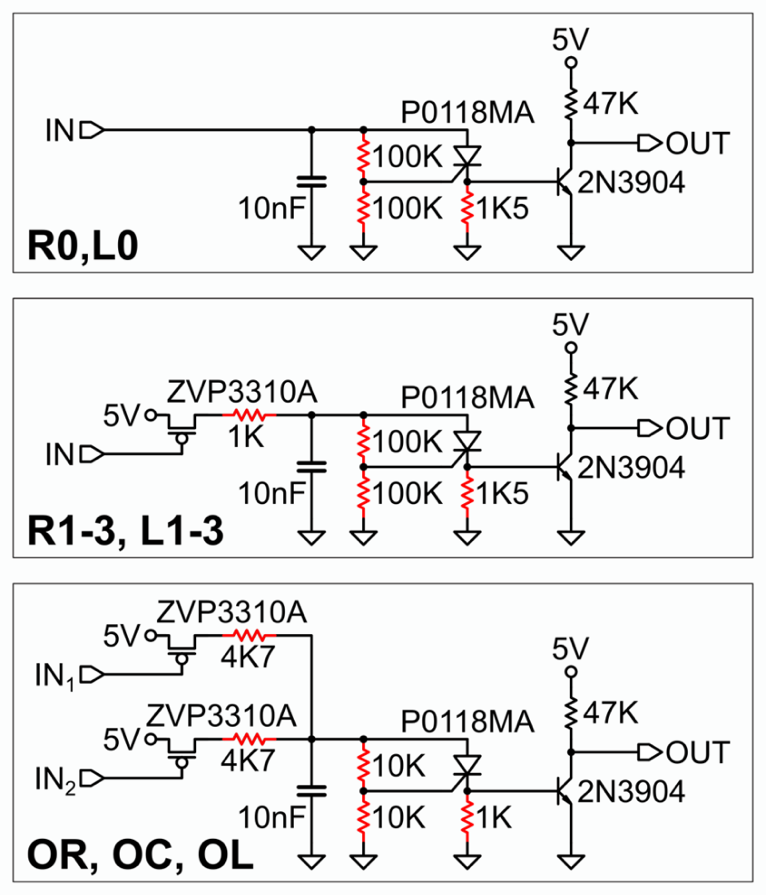


**Supplementary Figure 16.** Circuits used for the neurons. The components whose values are varied in different UCN blocks are indicated in red.

## Electrical measurements

To apply the input pulses to R0 and L0 we developed and ad-hoc pulse generator. We used 2 analog outputs of a National Instruments NI PCIe-6343 data acquisition board to generate voltage output pulses with amplitude adjustable between 0 and 1 V (width = 0.5ms). A LabVIEW-based control software generated these pulses with an adjustable delay (between them) between –20 us and +20 us.

The voltage pulses were converted to current pulses using the precision current source circuit proposed in Fig.7 of [1]. As instrumentation amplifier we used an LT1168 (instead of LT1167). As operational amplifier, we used a TL084 (instead of LT1464). R1 = 1kOhm, and RG was left open (gain = 1).

To measure the circuit signals we used a PicoScope 2205A MSO PC oscilloscope (2 analog inputs, 16 digital inputs).

Reference [1]: Strong A, “The LT1167: Precision, Low Cost, Low Power Instrumentation Amplifier Requires a Single Gain-Set Resistor”, Linear Technology Magazine, May 1998.
